# Effect of musculature on mortality, a retrospective cohort study

**DOI:** 10.1186/s12885-022-09751-6

**Published:** 2022-06-22

**Authors:** Amy L. Shaver, Mary E. Platek, Anurag K. Singh, Sung Jun Ma, Mark Farrugia, Gregory Wilding, Andrew D. Ray, Heather M. Ochs-Balcom, Katia Noyes

**Affiliations:** 1 Department of Epidemiology and Environmental Health, School of Public Health and Health Professions, University at Buffalo, Buffalo, NY USA; 2 Department of Medical Oncology, Sidney Kimmel Cancer Center at Jefferson, Sidney Kimmel Medical College, 834 Chestnut Street, Philadelphia, PA 19107 USA; 3 Department of Radiation Medicine, Roswell Park Comprehensive Cancer Center, Buffalo, NY USA; 4 Department of Dietetics, D’Youville College, Buffalo, NY 14203 USA; 5 Department of Biostatistics, School of Public Health and Health Professions, University at Buffalo, Buffalo, NY USA; 6 Department of Epidemiology, Roswell Park Comprehensive Cancer Center, Buffalo, NY USA

**Keywords:** Head and neck cancer, Muscle density, Body composition, Mortality, Radiotherapy, CCRT

## Abstract

**Background:**

While often life-saving, treatment for head and neck cancer (HNC) can be debilitating resulting in unplanned hospitalization. Hospitalizations in cancer patients may disrupt treatment and result in poor outcomes. Pre-treatment muscle quality and quantity ascertained through diagnostic imaging may help identify patients at high risk of poor outcomes early. The primary objective of this study was to determine if pre-treatment musculature was associated with all-cause mortality.

**Methods:**

Patient demographic and clinical characteristics were abstracted from the cancer center electronic database (*n* = 403). Musculature was ascertained from pre-treatment CT scans. Propensity score matching was utilized to adjust for confounding bias when comparing patients with and without myosteatosis and with and without low muscle mass (LMM). Overall survival (OS) was evaluated using the Kaplan–Meier method and Cox multivariable analysis.

**Results:**

A majority of patients were male (81.6%), white (89.6%), with stage IV (41.2%) oropharyngeal cancer (51.1%) treated with definitive radiation and chemotherapy (93.3%). Patients with myosteatosis and those with LMM were more likely to die compared to those with normal musculature (5-yr OS HR 1.55; 95% CI 1.03–2.34; HR 1.58; 95% CI 1.04–2.38).

**Conclusions:**

Musculature at the time of diagnosis was associated with overall mortality. Diagnostic imaging could be utilized to aid in assessing candidates for interventions targeted at maintaining and increasing muscle reserves.

**Supplementary Information:**

The online version contains supplementary material available at 10.1186/s12885-022-09751-6.

## Introduction

The treatments and side effects for head and neck cancer (HNC) can be dramatic in patients undergoing concomitant chemoradiation therapy (CCRT). CCRT in those with advanced HNC can produce grade 3 or worse toxicities including: hematological toxicities (decreases in bone marrow and blood cell counts that can lead to anemia, bleeding or infection), gastrointestinal reactions (nausea and vomiting), dermatitis, and mucositis; the adverse effects of treatment can be so severe as to require unplanned hospitalizations and can lead to treatment delay or interruption [1]. A treatment delay or interruption may in turn lead to an avoidable death.

A routine part of both diagnosis and treatment for HNC patients receiving definitive radiation therapy (RT) for HNC is computed tomography (CT) imaging which captures measures of body composition including muscle mass and muscle density. Skeletal muscle density (SMD), as measured through CT, refers to the radiodensity of the muscle fibers as found in muscle tissue; muscle density is inversely proportional to the amount of fatty infiltration into the muscle tissue, or myosteatosis sometimes referred to as muscle quality. Muscle mass can be compared between patients after CT measurement by calculating the skeletal muscle index (SMI). Since CT is routinely performed on HNC patients receiving definitive RT, SMD and SMI could be ascertained more regularly without causing additional patient burden. By ascertaining SMD and SMI, it may be possible to identify patients at risk for complications and poor outcomes during and after cancer treatment. Cancer patients facing unplanned hospitalization during cancer care are at increased risk of moderate to severe fatigue, depression, and post-traumatic stress disorder and may have to delay or stop their cancer treatment [2].

The aim of the current study was to evaluate the relationship between pre-treatment musculature and all-cause mortality in HNC patients. We hypothesized that those with poor musculature would have higher risk of dying from all causes than those with normal musculature.

## Methods

### Study design and population

We conducted a retrospective cohort study including survival analysis on a sample of squamous cell HNC patients treated with definitive radiation therapy over 18 years of age at Roswell Park Comprehensive Cancer Center (RPCCC), a facility in Western New York between 2008 and 2017. Those without readable whole-body -CT scans of the third lumbar (L3) vertebral body were excluded. Those persons where contrast dye was utilized were also excluded as contrast dye has been shown to alter the reported density of muscle tissue [3]. Survival was ascertained through clinical follow‐up, electronic medical record search, and follow‐up phone calls to patients and family members. The Institutional Review Board at RPCCC approved the study.

### Marker measurement

Imaging software (SliceOmatic Software by TomoVision, version 5.0) was used to quantify the cross-sectional area of muscle (a measure of skeletal muscle mass) and adipose tissue (in centimeters squared) at L3. The imaging software allows for measurement of skeletal muscle, visceral adipose tissue, subcutaneous adipose tissue, and intermuscular adipose tissue through the use of tissue-specific Hounsfield Units (HU) ranges [4]. The L3 level is used when estimating body composition as the estimates of skeletal muscle mass were previously and extensively validated based on measurements taken from the slice at this level of the body [5]. Other validation studies have shown that estimates of other whole body volumes from the L3 level are valid including fat estimates [6]. A measure of skeletal muscle mass, skeletal muscle index (SMI) was created by adjusting muscle mass for patient height (calculated by dividing the muscle area at L3 by patient height in meters squared). This adjustment is completed to enable comparisons between subjects and to determine low muscle mass (LMM). Skeletal muscle radiodensity (SMD), as measured by the mean radiation attenuation in HU, was used as the measure of muscle density.

SliceOmatic was also used to quantify adipose tissue in centimeters squared at the L3 level using the same method as described above [4]. Total adipose tissue (TAT) area at L3 in cm^2^ was constructed through addition of visceral adipose tissue (VAT), subcutaneous adipose tissue (SAT) and intermuscular adipose tissue (IMAT) and each was reported.

### Covariates and confounders

Age was expressed in years and parameterized as a continuous variable. Sex was parameterized as a dichotomous variable. Primary tumor site was recorded and reported as oropharynx, laryngeal, and other. Smoking status was categorized as current (an active daily smoker), former (an individual who has quit smoking at some point in the past and is now smoke-free), or never smoker (an individual who has never smoked). HPV was categorized as positive, negative, or inapplicable. Number of comorbidities was captured and parameterized as continuous. Staging of the tumor was categorized according to AJCC staging. Treatment was reported as follows: radiation therapy alone or radiation therapy plus chemotherapy. Median age of 61 years was utilized during analysis. Muscle density was dichotomized as myosteatosis and normal according to BMI appropriate cut-offs for head and neck cancer as previously described excluding the requirement for ≥ 8% weight loss as this is not a consistent criterion [7, 8]. Myosteatosis based on low muscle radiodensity has been used extensively in the literature. Myosteatosis was defined as < 41 Hounsfield units (HU) for those with a BMI in the healthy or underweight range (≤ 24.9) and < 33 HU for those with a BMI in the overweight or obese range (≥ 25.0) [7, 9]. Low muscle mass (LMM) was defined as SMI < 41 cm^2^/m^2^ in females and SMI < 43 cm^2^/m^2^ in males if of a normal BMI (BMI < 25 kg/m^2^) and LMM was SMI < 53 cm^2^/m^2^ if BMI ≥ 25 kg/m^2^ as done in prior studies [7, 9]. Unplanned hospitalization within 3 months after completing RT was dichotomous (yes vs no). Overall survival (OS) was defined as time interval from diagnosis to last follow up or death by any cause.

### Statistical analysis

To compare categorical and continuous variables in patients with and without unplanned hospitalizations, Fisher’s exact tests and student’s t-tests were performed, as appropriate.

Cox multivariable regression analysis was performed to evaluate variables associated with OS, after adjusting for age, gender, race, tumor stage, tumor site, treatment type, human papilloma virus (HPV) status, comorbidity, alcohol intake, and smoking status. Kaplan–Meier and log-rank tests were also performed to analyze OS. Propensity score matching in patients for myosteatosis and LMM was performed to control for confounding bias. Matching characteristics included clinically relevant variables used for Cox multivariable regression analysis. Matching was based on nearest neighbor method in a 1:1 ratio with no replacement and a caliper distance of 0.2 of the standard deviation of the logit of the propensity score [10, 11].

All *p* values were two-sided and variables with *p* ≤ 0.05 were considered significant. Statistical analyses were performed using SAS (SAS Institute, Cary, NC) and R (R Project for Statistical Computing, version 4.0.2).

## Results

### Population characteristics at baseline

Data from 403 patients were analyzed for this study (flowchart of included subjects Supplemental Figure 1). Baseline demographics and physical characteristics of patients prior to matching were recorded (Table 1). Median follow up was 64.5 months (interquartile range 40.3–87.1). The average age of patients was 60.9 ± 10.3 years and the majority were white (89.6%) and male (81.6%). The average number of comorbidities was nearly 2 (2.2 ± 1.8). Most patients were former smokers (50.1%), current alcohol drinkers (56.6%) and overweight (mean BMI 27.8 ± 5.8).

**Fig. 1:**
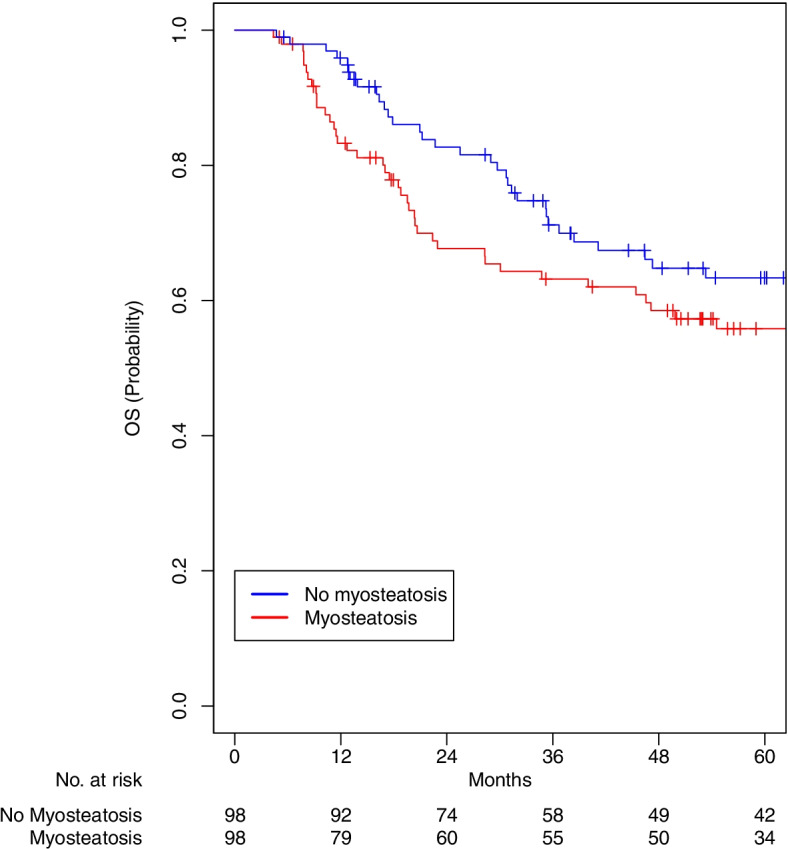
Overall Survival Myosteatosis compared to No Myosteatosis

**Table 1:** Patient characteristics overall and according to muscle density

Characteristic	All *n* = 403	Myosteatosis *n* = 150 (37.2)	Normal Musculature *n* = 253 (62.8)	*p*
Age (years)	60.9 (10.3)	65.2 (10.9)	58.4 (9.0)	< 0.0001
Sex				< 0.0001
Male	329 (81.6)	97 (64.7)	232 (91.7)	
Female	74 (18.4)	53 (35.3)	21 (8.3)	
Race				0.14
White	361 (89.6)	130 (86.7)	231 (91.3)	
BIPOC	42 (10.4)	20 (13.3)	22 (8.7)	
BMI (kg/m^2^)	27.8 (5.8)	25.3 (6.1)	29.3 (5.1)	< 0.0001
SMI (cm^2^/m^2^)	53.1 (11.7)	44.3 (8.4)	58.3 (10.1)	< 0.0001
VAT (cm^2^)	163.3 (96.3)	129.6 (88.1)	183.3 (95.5)	< 0.0001
SAT (cm^2^)	181.4 (94.7)	165.7 (102.9)	190.7 (88.5)	0.01
IMAT (cm^2^)	13.0 (8.1)	16.4 (9.3)	11.0 (6.5)	< 0.0001
TAT (cm^2^)	357.7 (164.5)	311.8 (168.0)	385.0 (156.4)	< 0.0001
SMD (HU)	38.6 (8.3)	30.5 (5.8)	43.4 (5.3)	< 0.0001
Low Muscle Mass				< 0.0001
Yes	135 (33.5)	85 (56.7)	50 (19.8)	
No	268 (66.5)	65 (43.3)	203 (80.2)	
Comorbidities	2.2 (1.8)	2.3 (1.8)	2.1 (1.9)	0.33
Tumor site				0.001
Oropharynx	206 (51.1)	61 (40.7)	145 (57.3)	
Larynx	100 (24.8)	51 (34.0)	49 (19.4)	
Other	32 (7.9	38 (25.3)	59 (23.3)	
AJCC stage				0.005
0-II	137 (34.0)	39 (26.0)	98 (38.7)	
III	100 (24.8)	40 (26.7)	60 (23.7)	
IV	166 (41.2)_	71 (47.3)	95 (37.6)	
HPV				< 0.0001
Positive	164 (40.7)	40 (26.7)	124 (49.0)	
Negative	96 (23.8)	48 (32.0)	48 (19.0)	
Inapplicable	143 (35.5)	62 (41.3)	81 (32.0)	
Treatment				0.001
RT only	27 (6.7)	18 (12.0)	9 (3.6)	
RT + Chemotherapy	376 (93.3)	132 (88.0)	244 (96.4)	
Smoking status				0.02
Current	110 (27.3)	50 (33.3)	60 (23.7)	
Former	202 (50.1)	76 (50.7)	126 (49.8)	
Never	91 (22.6)	24 (16.0)	67 (26.5)	
Alcohol consumption				0.41
Current	228 (56.6)	81 (54.0)	147 (58.1)	
Former	88 (21.8)	37 (24.7)	51 (20.2)	
Never	69 (17.1)	23 (15.3)	46 (18.2)	
Unknown	18 (4.5)	9 (6.0)	9 (3.6)	
Unplanned hospitalizations				0.12
None	308 (76.4)	121 (80.7)	187 (73.9)	
One	95 (23.6)	29 (19.3)	66 (26.1)	

*Abbreviations*: *AJCC* American Joint Committee on Cancer, *AMS* Altered mental status, *BMI* Body mass index, *HNC* Head and neck cancer, *HU* Hounsfield Units, *HPV* Human papilloma virus, *IMAT* Intermuscular adipose tissue, *kg* kilograms, *SAT* Subcutaneous adipose tissue, *SMD* Skeletal muscle density, *SMI* Skeletal muscle index, *TAT* Total adipose tissue, *VAT* Visceral adipose tissue

Data are presented as frequency (percent), mean (SD), or median (IQR)

A majority of patients had oropharyngeal cancer (51.1% overall) followed by laryngeal cancer (24.8%) and only just over one-third had HPV-associated tumors (40.7%). The distribution of stage from I-IV was as follows: 0.3% at stage 0, 4.2% at stage I, 29.5% at stage II, 24.8% at stage III, and 41.2% at stage IV. A vast majority of patients were treated with both definitive radiation and chemotherapy (93.3%), while the remainder of patients were treated with radiation alone (6.7%).

The average skeletal muscle density (SMD) among those with myosteatosis was 30.5 ± 5.8 HU and the average skeletal muscle index was 44.3 ± 8.4 cm^2^/m^2^; whereas among normal musculature these compositional measures were 43.4 ± 5.3 HU and 58.3 ± 10.1 cm^2^/m^2^, respectively. Total adipose tissue was different between the two groups (*p* < 0.0001) with the largest difference occurring in visceral adipose tissue (VAT). Within the baseline cohort, 135 patients (33.5%) had low muscle mass (LMM). Of those with LMM, 85 had co-occurring myosteatosis. There were 95 unplanned hospitalizations during the study period accounting for an incidence proportion of 23.6%.

### Mortality

During the study period, 180 persons died producing a mortality rate of 44.7%. Prior to matching, our Cox multivariable analysis showed both LMM (HR 1.25, 95% CI 0.89–1.75, *p* = 0.19) and myosteatosis (HR 1.14, 95% CI 0.81–1.60, *p* = 0.46) were not associated with OS.

**Fig. 2:**
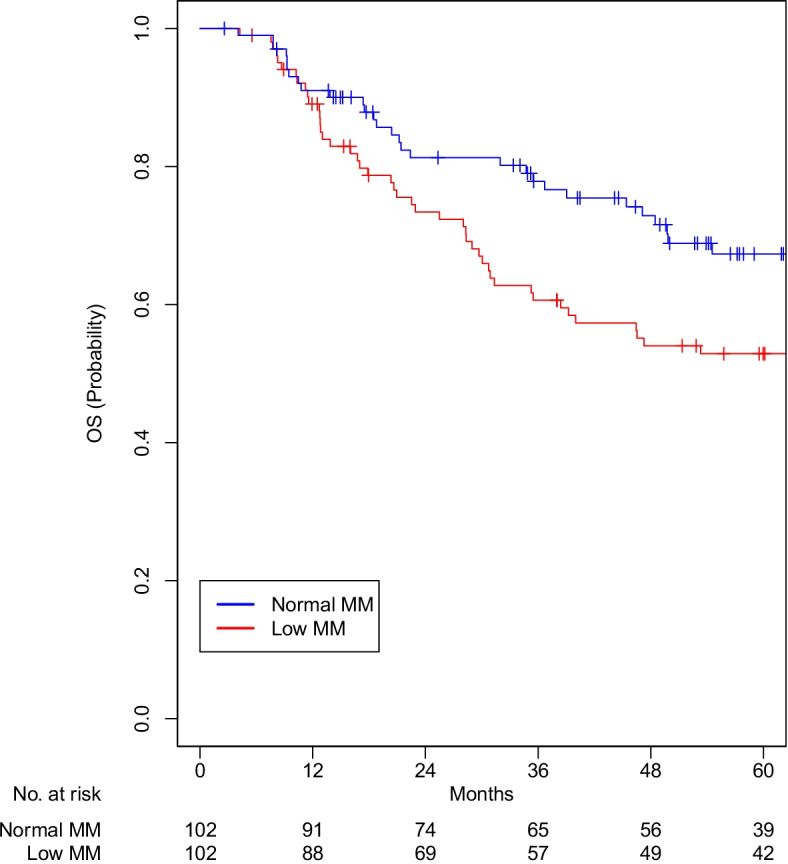
Overall Survival Low Muscle Mass compared to Normal Muscle Mass

After matching, all variables were well balanced (Table 2). A total of 98 and 102 matched pairs were identified for those with versus without myosteatosis and low versus normal muscle mass, respectively. Patients with myosteatosis were associated with worse OS (5-year OS 55.8% vs 63.4%; HR 1.55, 95% CI 1.03–2.34, *p* = 0.037; Fig. 1). Patients with LMM were associated with worse OS (5-year OS 52.9% vs 67.3%; HR 1.58, 95% CI 1.04–2.38, *p* = 0.032; Fig. 2).

**Table 2:** Characteristics of matched pairs

	**Myosteatosis *n* = 98**		**No Myosteatosis *n* = 98**			**LMM *n* = 102**		**Normal MM *n* = 102**		
	**N**	**%**	**N**	**%**	***p***	**N**	**%**	**N**	**%**	***p***
**Age**					**0.88**					**1**
< 61	62	63.3	64	65.3		68	66.7	69	67.6	
> = 61	36	36.7	34	34.7		34	33.3	33	32.4	
**Sex**					**0.60**					**0.85**
Female	22	22.4	18	18.4		18	17.6	16	15.7	
Male	76	77.6	80	81.6		84	82.4	86	84.3	
**Race**					**0.65**					**0.81**
White	86	87.8	89	90.8		94	92.2	92	90.2	
BIPOC	12	12.2	9	9.2		8	7.8	10	9.8	
**Comorbidities**					**0.96**					**0.86**
None	16	16.3	18	18.4		17	16.7	19	18.6	
1	25	25.5	27	27.6		25	24.5	27	26.5	
2	16	16.3	14	14.3		14	13.7	16	15.7	
3 +	41	41.8	39	39.8		46	45.1	40	39.2	
**Tumor site**					**0.79**					**0.66**
Oropharynx	44	44.9	46	46.9		48	47.1	53	52.0	
Larynx	31	31.6	33	33.7		32	31.4	26	25.5	
Other	23	23.5	19	19.4		22	21.6	23	22.5	
**AJCC stage**					**0.34**					**0.98**
0-II	28	28.6	32	32.7		30	29.4	32	31.4	
III	19	19.4	25	25.5		24	23.5	24	23.5	
IV	51	52.0	41	41.8		48	47.1	46	45.1	
**HPV**					**0.81**					**0.68**
Positive	21	21.4	23	23.5		22	21.6	26	25.5	
Negative	29	29.6	32	32.7		35	34.3	37	36.3	
N/A	48	49.0	43	43.9		45	44.1	39	38.2	
**Treatment**					**1**					**1**
RT only	7	7.1	8	8.2		6	5.9	6	5.9	
Chemoradiation	91	92.9	90	91.8		96	94.1	96	94.1	
**Smoking**					**0.98**					**0.80**
Current	20	20.4	20	20.4		22	21.6	26	25.5	
Former	45	45.9	47	48.0		50	49.0	48	47.1	
Never	33	33.7	31	31.6		30	29.4	28	27.5	
**Alcohol**					**0.92**					**0.75**
Current	18	18.4	18	18.4		19	18.6	16	15.7	
Former	20	20.4	21	21.4		20	19.6	26	25.5	
Never	56	57.1	57	58.2		60	58.8	58	56.9	
N/A	4	4.1	2	2.0		3	2.9	2	2.0	
**Muscle mass**					**1**					**NA**
Normal	49	50.0	48	49.0		0	0.0	102	100.0	
Low	49	50.0	50	51.0		102	100.0	0	0.0	
**Myosteatosis**					**NA**					**1**
No	0	0.0	98	100.0		53	52.0	53	52.0	
Yes	98	100.0	0	0.0		49	48.0	49	48.0	

*Abbreviations*: *AJCC* American Joint Committee on Cancer, *BIPOC* Black Indigenous People of Color, *LMM* Low muscle mass, *MM* Muscle mass, *RT* Radiation therapy

Data are presented as frequency (percent), mean (SD)

## Discussion

The results of the study indicated that baseline musculature (both myosteatosis and low muscle mass) was predictive of all-cause mortality in this cohort of HNC patients. Low muscle mass has been tied to chemotoxicity through a number of mechanisms. Chemotherapy and radiation can cause muscle wasting via inflammation and activation of the NF-κB pathway [12, 13]. Those patients with less dense muscle or, less muscle mass at the beginning of therapy may be less likely to tolerate full therapy and therefore more likely to succumb to their cancer [14].

The study population was in accord with HNC populations typical of the United States: the study population was predominately male, non-Hispanic white, with an oropharyngeal primary tumor site [15]. Our measure of SMD was slightly higher than a prior study of myosteatosis and sarcopenia in HNC patients (38.6 vs 30.5 HU at baseline) [7]. Our measure of SMD was in accord with other studies of different cancer patients prior to treatment [16, 17]. A systematic review found a wide range of prevalence in sarcopenic and low muscle mass definitions. Pre-treatment prevalence ranged from 6.6–70% in HNC patients [18]. The review found that low muscle mass was associated with decreased overall survival but that more studies were needed to verify the findings.

A recent prospective cohort study by Thureau et al. examined the relationship between pre-treatment sarcopenia (determined solely through CT evaluation at the L3 level) and both treatment-related toxicities and overall survival [19]. The current study was in accord with the prospective cohort study’s findings. The Thureau et al. study found that although sarcopenia did not have an association with treatment related toxicity there was a significant association with overall survival HR 1.9 (95% CI 1.1, 3.25) which is in line with the findings of the current study.

A similarly sizedstudy (matched sample *n* = 100 vs *n* = 99) by Findlay et al*.* indicated that treatment completion was similar for those HNC patients with and without pre-treatment myosteatosis while including a much higher percentage of stage IV cancers (62%) [7]. The same study found not significant association between pre-treatment myosteatosis and unplanned hospitalization (OR 3.45; 95% CI 0.93, 12.64; *p* = 0.063). The Findlay et al. study performed a survival analysis and also found similar associations to the current study between overall survival, baseline LMM (HR 3.87; 95% CI 1.22, 12.24; *p* = 0.02) and myosteatosis (HR 8.86; 95% CI 1.12, 69.88; *p* = 0.038).

A retrospective cohort study was conducted at the University Medical Center Utrecht on locally advanced HNC patients treated with chemoradiation. The study was conducted in a similar timeframe to this study from 2012 to 2018. Chargi et al. also found that low skeletal muscle mass at diagnosis was prognostic for overall survival (HR 2.1; 95% CI 1.1–4.1; *p* = 0.03).

Muscle density and myosteatosis are still relatively new areas of exploration in HNC and so comparable studies are limited. A study by Murnane et al. examined the rate of overall survival and complications following radical surgery in oesophageal and gastric cancer patients. Those with myosteatosis compared to normal musculature had a reduced 5 year overall (54.1 vs. 83%, *p* = 0.004) and disease-free (55.2 vs. 87.2%, *p* = 0.007) survival.

A study by Charette et al*.* performed a post-hoc analysis of two clinical trials on colorectal cancer patients [20]. The post-hoc analysis indicated that myosteatosis was indicative of poor survival which is similar to the findings of this study. Charette et al*.* also found that the factor with the most negative impact on survival was visceral adipose tissue and those are the persons in the current study who were hospitalized at a higher percent.

A recent study by Schaffler-Schaden et al*.* failed to find a significant association between visceral adiposity, BMI, myosteatosis, and complications following surgery with curative intent in colorectal cancer patients [21]. The Schaffler-Schaden et al*.* study indicated that in the non-obese population the only statistically significant predictor was lean muscle mass. It is possible that the effect of myosteatosis is different in the non-obese population.

The study had a number of strengths. The study was a cohort design allowing for the exposure to be ascertained prior to the outcome. Only patients with imaging of L3 were included which allowed for consistency in the measurement of body composition parameters and served to decrease measurement bias. Patient scans were only be used if they were full-body CT scans thus improving rigor and reproducibility. All patients were managed by one radiation oncologist which allowed for consistency in care decisions.

The study also had some limitations. The study contains patients with multiple cancer sites, however an attempt was made to control for this by including it in the final model. The study is also a single-center study and so its findings may not be broadly applicable.

The pre-treatment prevalence of myosteatosis and low muscle mass was 37.2 and 33.5%, respectively. Both myosteatosis and low muscle mass were significantly associated with mortality. The question of musculature and treatment effects requires more study so as to determine an appropriate and feasible response. Diagnostic and planning imaging could potentially be utilized to give early and specific body composition and malnutrition information to the healthcare team in an effort to improve outcomes.

## Supplementary Information

**Additional file 1:** **Supplemental Figure 1.** Inclusion Flow Chart.

## Data Availability

Data cannot be shared publicly because of protected health information. Data are available from the respective center Institutional Data Access / Ethics Committee (contact via email) for researchers who meet the criteria for access to confidential data. Please contact RSPAdmin@RoswellPark.org regarding the head and neck database under EDR-103707.

## Abbreviations

AJCC: American Joint Committee on Cancer
BMI: Body mass index
CCRT: Concomitant chemoradiation therapy
CT: Computed tomography
HNC: Head and neck cancer
HU: Hounsfield Units
HPV: Human papilloma virus
IMAT: Intermuscular adipose tissue
Kg: Kilograms
LMM: Low Muscle Mass
MM: Muscle Mass
OR: Odds ratios
RPCCC: Roswell Park Comprehensive Cancer Center
SAT: Subcutaneous adipose tissue
SMD: Skeletal muscle density
SMI: Skeletal muscle index
TAT: Total adipose tissue
VAT: Visceral adipose tissue

## References

[1] Guan J, Zhang Y, Li Q (2016). A meta-analysis of weekly cisplatin versus three weekly cisplatin chemotherapy plus concurrent radiotherapy (CRT) for advanced head and neck cancer (HNC). Oncotarget.

[2] Newcomb RA, Nipp RD, Waldman LP (2020). Symptom burden in patients with cancer who are experiencing unplanned hospitalization. Cancer.

[3] Paris MT, Furberg HF, Petruzella S (2018). Influence of contrast administration on computed tomography-based analysis of visceral adipose and skeletal muscle tissue in clear cell renal cell carcinoma. JPEN J Parenter Enteral Nutr.

[4] Prado CMM, Heymsfield SB (2014). Lean tissue imaging: a new era for nutritional assessment and intervention. JPEN J Parenter Enteral Nutr.

[5] Vangelov B, Bauer J, Kotevski D (2022). The use of alternate vertebral levels to L3 in computed tomography scans for skeletal muscle mass evaluation and sarcopenia assessment in patients with cancer: a systematic review. Br J Nutr.

[6] Shen W, Punyanitya M, Wang Z (1985). Total body skeletal muscle and adipose tissue volumes: estimation from a single abdominal cross-sectional image. J Appl Physiol.

[7] Findlay M, Brown C, De Abreu LR (2020). Sarcopenia and myosteatosis in patients undergoing curative radiotherapy for head and neck cancer: impact on survival, treatment completion, hospital admission and cost. J Hum Nutr Diet..

[8] Stretch C, Aubin JM, Mickiewicz B (2018). Sarcopenia and myosteatosis are accompanied by distinct biological profiles in patients with pancreatic and periampullary adenocarcinomas. PLoS One.

[9] Su H, Ruan J, Chen T (2019). CT-assessed sarcopenia is a predictive factor for both long-term and short-term outcomes in gastrointestinal oncology patients: a systematic review and meta-analysis. Cancer Imaging.

[10] Lunt M (2013). Selecting an appropriate caliper can be essential for achieving good balance with propensity score matching. Am J Epidemiol.

[11] Austin PC (2011). Optimal caliper widths for propensity-score matching when estimating differences in means and differences in proportions in observational studies. Pharm Stat.

[12] Damrauer JS, Stadler ME, Acharyya S (2018). Chemotherapy-induced muscle wasting: association with NF-κB and cancer cachexia. Eur J Transl Myol.

[13] Gilliam LA, St Clair DK (2011). Chemotherapy-induced weakness and fatigue in skeletal muscle: the role of oxidative stress. Antioxid Redox Signal.

[14] Pin F, Couch ME, Bonetto A (2018). Preservation of muscle mass as a strategy to reduce the toxic effects of cancer chemotherapy on body composition. Curr Opin Support Palliat Care.

[15] Siegel RL, Miller KD, Jemal A (2020). Cancer statistics, 2020. CA Cancer J Clin.

[16] Dijksterhuis WPM, Pruijt MJ, van der Woude SO (2019). Association between body composition, survival, and toxicity in advanced esophagogastric cancer patients receiving palliative chemotherapy. J Cachexia Sarcopenia Muscle.

[17] Shachar SS, Deal AM, Weinberg M (2017). Body composition as a predictor of toxicity in patients receiving anthracycline and taxane-based chemotherapy for early-stage breast cancer. Clin Cancer Res.

[18] Findlay M, White K, Lai M (2020). The Association between computed tomography-defined sarcopenia and outcomes in adult patients undergoing radiotherapy of curative intent for head and neck cancer: a systematic review. J Acad Nutr Diet.

[19] Thureau S, Lebret L, Lequesne J (2021). Prospective evaluation of sarcopenia in head and neck cancer patients treated with radiotherapy or radiochemotherapy. Cancers (Basel).

[20] Charette N, Vandeputte C, Ameye L (2019). Prognostic value of adipose tissue and muscle mass in advanced colorectal cancer: a post hoc analysis of two non-randomized phase II trials. BMC Cancer.

[21] Schaffler-Schaden D, Mittermair C, Birsak T (2020). Skeletal muscle index is an independent predictor of early recurrence in non-obese colon cancer patients. Langenbecks Arch Surg.

