# Supplementary figures and images for: Effect of musculature on mortality, a retrospective cohort study
Source: BMC Cancer. 2022 Jun 22;22:688. doi: 10.1186/s12885-022-09751-6 (PMC9214966; doi:10.1186/s12885-022-09751-6)

**Supplemental Figure 1. Inclusion Flow Chart**

**
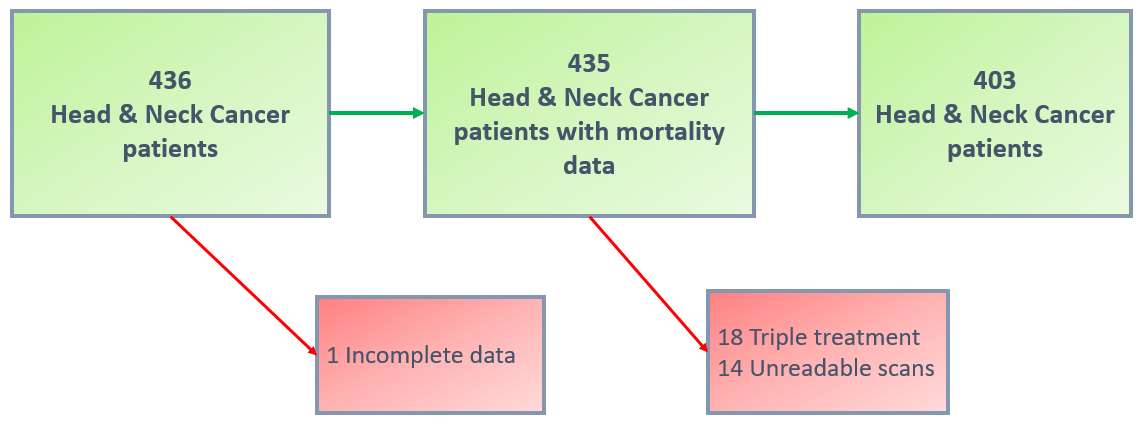
**

Supplement: Supplementary file 1 — Additional file 1: Supplemental Figure 1. Inclusion Flow Chart. [file 12885_2022_9751_MOESM1_ESM.docx]
